# Immunodeficiency: A Protective Factor for COVID-19?

**DOI:** 10.7759/cureus.23094

**Published:** 2022-03-12

**Authors:** Zain AlShanableh, Mohammad Haidous, Krista M Wong, Mohamad Al-Saed, Basel Altaqi

**Affiliations:** 1 Internal Medicine, Saint Vincent Charity Medical Center, Cleveland, USA

**Keywords:** ards, cytokine storm, cd4+ lymphocytopenia, immunodeficiency, covid-19

## Abstract

Coronavirus disease 2019 (COVID-19) is an infection that involves the respiratory tract and is attributed to severe acute respiratory syndrome coronavirus 2. While most people develop mild or uncomplicated illness, approximately 15% develop a severe disease that requires oxygen support, and 5% develop a critical disease. While immunodeficiency is reported as a risk factor for COVID-19, we present a patient with idiopathic CD4+ lymphocytopenia who developed severe COVID-19 with an unexpected clinical course and complete recovery.

## Introduction

Towards the end of 2019, several cases of pneumonia emerged in Wuhan, China, and were attributed to a new form of coronavirus. It expeditiously spread globally resulting in a pandemic. The disease, coronavirus disease 2019 (COVID-19), was designated a pandemic by the World Health Organization in February of 2020. COVID-19 is an infection that involves the respiratory tract and is attributed to severe acute respiratory syndrome coronavirus 2 (SARS-CoV-2). This coronavirus is genetically identical to the coronavirus that caused the SARS outbreak of 2003, SARS-CoV-1 [1]. While immunodeficiency is reported as a risk factor for COVID-19, we present a patient with idiopathic CD4+ lymphocytopenia who developed severe COVID-19 with an unexpected clinical course and complete recovery raising the possibility of immunodeficiency being a protective factor.

## Case presentation

**Figure 1:**
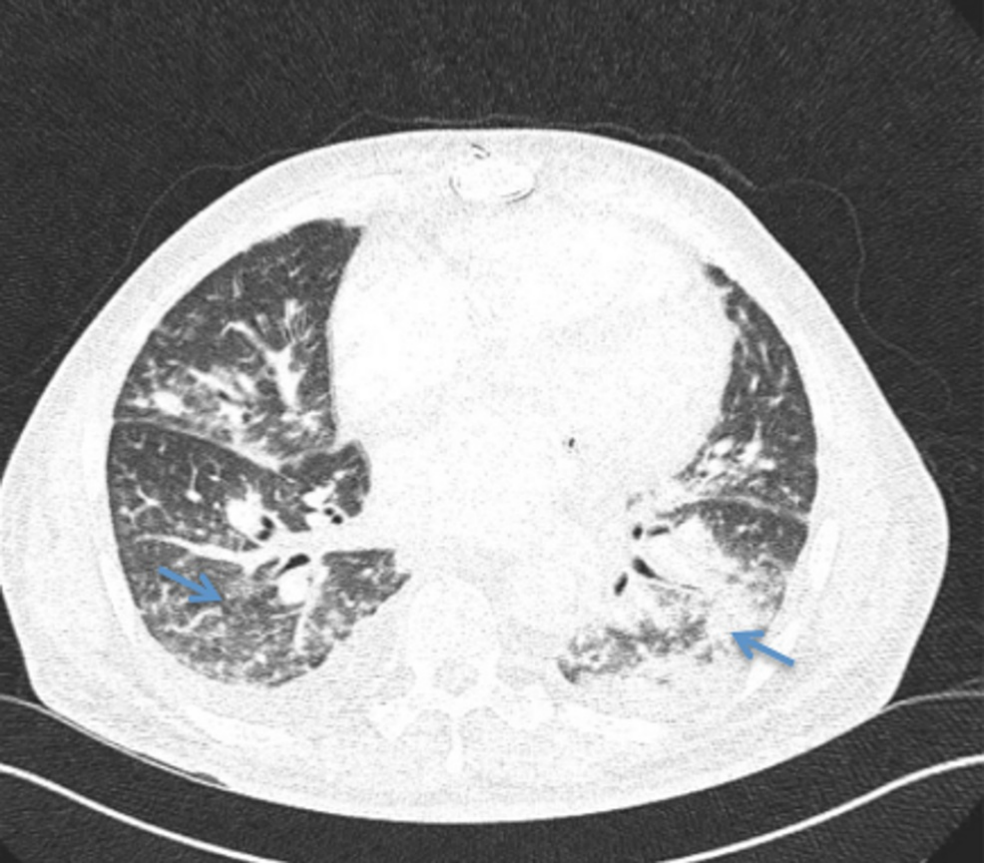
Computed tomography scan images of the chest. Diffuse nodular infiltrates (blue arrows).

A 54-year-old male with a medical history of immunoglobulin (Ig)A deficiency, idiopathic CD4+ lymphocytopenia, immune-thrombocytopenic purpura status post-splenectomy, autoimmune hemolytic anemia, infectious endocarditis status post-triple valve replacement, and atrial fibrillation presented with cough, fever, and pleuritic chest pain for five days. On examination, he was febrile and tachycardic with a temperature of 38.2°C, a heart rate of 118 beats per minute, and saturating 96% on 3 L of oxygen via a nasal cannula. Lung auscultation revealed diffuse crackles bilaterally. The remaining examination was unremarkable. Labs revealed elevated inflammatory markers with ferritin 9,922 ng/mL, lactate dehydrogenase 725 U/L, C-reactive protein 17.4 mg/L, procalcitonin 0.22 ng/mL, and lactic acid 1.4 mmol/L. Computed tomography of the chest revealed diffuse nodular infiltrates, more pronounced in the lower lobes (Figure 1).

Immunology obtained five months before admission for other reasons revealed absolute CD4 count of 370 cells/µL, IgG 1,210 mg/dL, IgA <5 mg/dL, IgM 61 mg/dL, and IgE <2 IU/mL (Table 1). The patient tested positive for SARS-CoV-2 and, at that time, was started on hydroxychloroquine and azithromycin. He required oxygen supplementation via nasal cannula but not invasive ventilation, that is, bilevel positive airway pressure, continuous positive airway pressure, or endotracheal intubation. Despite having elevated inflammatory markers, he did not have clinical evidence of a cytokine storm. His symptoms resolved, repeat swabs were negative, and he was subsequently discharged off oxygen.

**Table 1:** Laboratory findings. *Procalcitonin of ≤0.5 ng/mL: sepsis is not likely, but local infection is possible; procalcitonin of 0.5-2 ng/mL: sepsis is possible, but other conditions are known to elevate procalcitonin as well; procalcitonin of >2.0 ng/mL: sepsis is likely unless other causes are known; procalcitonin of ≥10 ng/mL: important systemic inflammatory response, almost exclusively due to severe bacterial sepsis or septic shock.

Laboratory work	Value	Reference range
Ferritin	9,922 ng/mL	26–388 ng/mL
Lactate dehydrogenase	725 U/L	82–246 U/L
C-reactive protein	17.4 mg/L	0.0–3.0 mg/L
Procalcitonin	0.22 ng/mL	See below*
Lactic acid	1.4 mmol/L	0.4–2.0 mmol/L
Absolute CD4 count	370 cells/µL	490–1,740 cells/µL
Immunoglobulin G	1,210 mg/dL	700–1,600 mg/dL
Immunoglobulin A	<5 mg/dL	90-386 mg/dL
Immunoglobulin E	<2 IU/mL	6–495 IU/mL
Immunoglobulin M	61 mg/dL	20–172 IU/mL

## Discussion

COVID-19 is an infection that involves the respiratory tract and is attributed to SARS-CoV-2 that emerged in 2019. Genetic studies have revealed that SARS-CoV-2 is a beta coronavirus that is closely related to the SARS virus. While the majority of people develop mild or uncomplicated illness, around 15% develop a disease that is severe requiring oxygen supplementation, and another 5% have a critical illness with complications such as acute respiratory distress syndrome (ARDS), respiratory failure, thromboembolism, sepsis and septic shock, and/or multiorgan failure, including acute cardiac injury and kidney injury [2].

CD4+ T-cells play a crucial role in the immune system and its response to various pathogens. Antigen recognition and presentation by antigen-presenting cells (APC) results in the differentiation of naïve CD4+ T-cells into effectors. Three major subtypes of effector CD4+ T-cells have been recognized: T-helper 1 (Th1), Th2, and Th17 cells. Immunity against intracellular viral and bacterial pathogens is provided by Th1 cells via methods that include activation of macrophages, “help” for B cells’ antibody production, and release of other pro-inflammatory mediators. Upon interaction of naive T-cell with APCs, Th0 expresses interleukin (IL)-12 receptors and produces IL-12 resulting in autostimulation and differentiation into Th1 cells, which, in turn, produce interferon-gamma (IFN-γ) and tumor necrosis factor-alpha (TNF-α), resulting in the recruitment and activation of macrophages or cytotoxic T-lymphocytes (CTLs) and release of other cytokines. Th/c17 cells are differentiated by IL-1β, IL-6, and transforming growth factor beta. Th17-cells secrete IL-17, which activates innate-immune cells, recruits neutrophils, and releases other cytokines. IL-17 also contributes to organ-specific autoimmunity and pathogenic inflammatory conditions [3].

In severe coronavirus pneumonia, rapid virus replication followed by a large number of inflammatory cell infiltration and cytokine storm may lead to ARDS and death. Cytokine storm is a hyperinflammatory state characterized by fulminant multiorgan failure and elevation of cytokine levels due to persistent activation of macrophages and CTLs [4]. A study showed that COVID-19 is associated with cytokine elevation that is reminiscent of secondary hemophagocytic lymphohistiocytosis [5].

Given that our patient has low CD4 counts, he was unable to initiate the T-cell differentiation cascade and therefore unable to mount a severe inflammatory immune response. Dexamethasone and tocilizumab are currently being used for the treatment of COVID-19. Dexamethasone binds glucocorticoid receptors forming a complex that binds to specific DNA sites resulting in stimulation and suppression of varied gene transcriptions. It can inhibit the production of pro-inflammatory cytokines such as IL-1, IL-2, IL-6, IL-8, TNF-α, IFN-γ, and vascular endothelial growth factor, which are linked to SARS-CoV-2 severity [6]. Tocilizumab is a monoclonal antibody that binds IL-6 receptors, thus blocking IL-6 signaling and its mediated inflammatory response [4]. The mechanism of action of these two drugs further supports that immunodeficiency could be protective to a certain extent.

There have been two other studies that report cases of primary immunodeficiency (PID) in adults who developed COVID-19. Soresina et al. reported two patients with X‐linked agammaglobulinemia (XLA) aged 34 and 26 years who developed COVID‐19 but never required oxygen ventilation or intensive care [7]. Another case series from Italy described seven patients with PID and concluded that patients with agammaglobulinemia had mild symptoms in comparison to patients with common variable immunodeficiency [8]. However, the previously mentioned cases described B-cell dysfunction. To our knowledge, this is the only reported patient of primary CD4 deficiency who developed severe COVID-19 but no critical illness and had a complete recovery.

## Conclusions

SARS-CoV-2 is a novel virus and several retrospective studies are yet to be done to fully understand the disease it causes and its mechanism. Our reported case was of particular interest as our patient was known to be immunodeficient, yet did not develop critical illness and had an exceptional clinical course with complete recovery, raising the possibility of immunodeficiency being a protective factor. Larger studies are yet to be performed to determine whether immunodeficiency is a protective factor or, paradoxically, a risk factor for COVID-19. Furthermore, understanding the molecular level of the disease will help us propose further treatment options.
